# Supplementary material for: Household determinants of animal and human fecal contamination on floors and hands in northwestern coastal Ecuador
Source: medRxiv. 2026 Aug 6:2026.08.04.26359736. Preprint. [Version 1] doi: 10.64898/2026.08.04.26359736 (PMC13484808; doi:10.64898/2026.08.04.26359736)

Supporting Information

Household determinants of animal and human fecal contamination on floors and hands in northwestern coastal Ecuador

Viviana Albán,[1*] Kelsey J. Jesser,[1,2] Aldo Lobos,[3] Javier Gallard-Góngora,[3] April M. Ballard,[4] Gwenyth O. Lee,[5] Joseph N.S. Eisenberg,[6] Erica R. Fuhrmeister,[1,7] Joan A. Casey,[1] Gabriel Trueba,[8] Christine Fagnant-Sperati,[1] Valerie J. Harwood,[3] Karen Levy,[1] and the ECoMiD Authorship Group†

[1] Department of Environmental and Occupational Health Sciences, University of Washington, Seattle, Washington, USA

[2] Department of Environmental Health Sciences, University of Georgia, Athens, Georgia, USA

[3] Department of Integrative Biology, University of South Florida, Tampa, Florida, USA

[4] Department of Population Health Sciences, Georgia State University School of Public Health, Atlanta, GA, USA

[5] Rutgers Global Health Institute and Department of Biostatistics and Epidemiology, Rutgers University, New Brunswick, New Jersey, USA

[6] Department of Epidemiology, University of Michigan School of Public Health, Ann Arbor, Michigan, USA

[7] Department of Civil and Environmental Engineering, University of Washington, Seattle, Washington, USA

[8] Instituto de Microbiología, Colegio de Ciencias Biológicas y Ambientales, Universidad San Francisco de Quito, Quito, Ecuador

†ECoMiD Authorship Group: William Cevallos, Adriana Lupero, Mauricio Ayoví, Stuart Torres Ayala and Lesly Simbaña Vivanco

Corresponding author's email address: klevyx@uw.edu

Table of Contents

Table S1: MST qPCR reaction primers, probes sequences 3

Table S2: Procedures for LOD, LOQ, DQ, DNQ and ND determination following the MIQE guidelines. 4

Table S4: Associations between animal-related variables and presence/absences of any animal MST marker 5

Table S5: Associations between animal-related variables and mean abundance of all animal MST markers. 6

Table S6: Associations between household and WASH-related variables and presence/absences of human HF183 MST marker. 7

Table S7: Associations between household and WASH-related variables and abundance of human HF183 MST marker. 8

Figure S1: Known groups construct validity of the modified binary FECEZ Enteropathogens 9

Figure S2. Directed acyclic graph (DAG). Illustrating the hypothesized relationships between environmental risk factors, covariates, and MST marker presence on floors, child hands, and maternal hands. 10

Figure S3: Canine and Avian MST markers (presence/absence) associations 11

Figure S4: Canine and Avian MST markers (abundance) associations 12

Table S1: MST qPCR reaction primers, probes sequences and running conditions. F: forward; R: reverse; P: probe; IAC: Internal Amplification Control.

MST marker	Target microorganism	Host	Primer and probe sequences (5'-3')	Running conditions
HF183	(*B. dorei 16S gene*)	Human	F: ATCATGAGTTCACATGTCCG<br>BacR287: CTTCCTCTCAGAACCCCTATCC<br>HF183 P:[FAM]CTAATGGAACGCATCCC[MGB]<br>IAC P: [VIC]AACACGCCGTTGCTACA[MGB]	10 min at 95°C; 40 cycles of 15 s at 95°C and 1 min at 60°C
DG37	(*Bacteroides*)	Canine	F: TTTTCTCCCACGGTCATCTG<br>R: CTTGGTTATGGGCGACATTG<br>P: [FAM]TGAACGTTTAAAGGAGCAGGTGGCAG[TAMRA]	15 min at 95°C; 45 cycles of 15 s at 95°C and 1 min at 60°C
GFD	(*Helicobacter* spp.)	Avian	F: TCGGCTGAGCACTCTAGGG<br>R: GCGTCTCTTTGTACATCCCA	10 min at 95°C; 40 cycles of 15 s at 95°C, 30 s at 57°C, and 30 s at 72°C
Rum2Bac	(*Bacteroidales*)	Ruminant	F: ACAGCCCGCGATTGATACTGGTAA<br>R: CAATCGGAGTTCTTCGTGAT<br>P: [FAM] ATGAGGTGGATGGAATTCGTGGTGT[BHQ-1]	10 min at 95°C 40 cycles of 15 s at 95°C and 1 min at 60°C
Pig2Bac	(*Bacteroidales*)	Swine	F: GCATGAATTTAGCTTGCTAAATTTGAT<br>R: ACCTCATACGGTATTAATCCGC<br>P: [VIC]TCCACGGGATAGCC [NFQ-MGB]	10 min at 95°C followed by 40 cycles of 15 s at 95°C and 1 min at 60°C

Table S2: Procedures for LOD, LOQ, DQ, DNQ and ND determination following the MIQE guidelines.

qPCR item	Description
Limit of Detection (LOD)	The lowest standard on the curve at which at least 2 of 3 replicate reactions amplify.
Limit of Quantification (LOQ)	The lowest standard on the curve at which all 3 replicate reactions amplify, with acceptable precision<br>CtLOQ = CtLOD standard – 2σ, where σ is the standard deviation of Ct values across replicate runs of the LOD standard.
Detectable Quantifiable (DQ)	Both duplicates amplified, were within 2 Ct values of each other, and fell within the quantifiable range (at or below CtLOQ). The mean of the two duplicate Ct values was assigned.
Detectable but not quantifiable (DNQ)	Both duplicates amplified, but above CtLOQ (i.e., between the LOD and LOQ). The Ct value of the LOD standard was assigned.
Not detected (ND)	Neither duplicate amplified, or only one of the two amplified. Half the Ct value of the LOD standard was assigned in either case.

Table S4: Associations between animal-related variables and presence/absences of any animal MST marker. Prevalence ratios (PRs), 95% confidence intervals (CIs), and p-values were calculated using a modified Poisson regression model with a log link and robust standard errors. P-values are based on z-statistics computed as the coefficient divided by its robust standard error, assuming a normal distribution.

Animal-related Variables	Any sample			Floors			Child hands			Maternal hands		
	PR	[95% CIs]	p-value	PR	[95% CIs]	p-value	PR	[95% CIs]	p-value	PR	[95% CIs]	p-value
**Animal Ownership**												
Animals spend time outside HH<br>***Ref: Animals do not spend time outside***	1.02	[0.76, 1.36]	0.92	1.34	[0.88, 2.03]	0.18	0.69	[0.41, 1.14]	0.14	1.33	[0.70, 2.56]	0.39
Animals spend time inside HH<br>***Ref: Animals do not spend time outside***	1.10	[0.79, 1.53]	0.58	1.03	[0.64, 1.67]	0.89	0.79	[0.41, 1.54]	0.49	1.36	[0.68, 2.75]	0.38
Animals owned (>5)<br>***Ref: No animals owned***	1.13	[0.82, 1.57]	0.46	1.67	[1.05, 2.65] *	0.03	0.83	[0.42, 1.63]	0.58	1.47	[0.69, 3.14]	0.31
Animals owned (1-5)<br>***Ref: No animals owned***	1.10	[0.80, 1.50]	0.57	1.46	[0.93, 2.30]	0.10	0.67	[0.37, 1.19]	0.17	1.06	[0.53, 2.13]	0.87
Animals owned<br>***Ref: No animals owned***	1.11	[0.85, 1.45]	0.46	1.53	[1.04, 2.26] *	0.03	0.72	[0.44, 1.18]	0.19	1.18	[0.64, 2.18]	0.59
**Animal Feces**												
Animal feces-Other management<br>***Ref: No animal feces***	0.99	[0.58, 1.67]	0.96	1.50	[0.78, 2.89]	0.22	0.75	[0.25, 2.21]	0.60	1.20	[0.43, 3.36]	0.73
Animal feces left sometime/not removed<br>***Ref: No animal feces***	0.86	[0.56, 1.33]	0.50	0.78	[0.36, 1.67]	0.52	0.87	[0.43, 1.76]	0.70	2.31	[1.11, 4.81] *	0.03
Animal feces removed as soon as seen<br>***Ref: No animal feces***	0.99	[0.72, 1.36]	0.96	1.19	[0.77, 1.84]	0.43	0.63	[0.32, 1.26]	0.19	1.36	[0.64, 2.88]	0.42
Animal feces outside HH<br>***Ref: No animal feces***	0.96	[0.73, 1.25]	0.75	1.14	[0.77, 1.67]	0.52	0.72	[0.44, 1.18]	0.19	1.53	[0.85, 2.76]	0.16
Animal feces inside HH<br>***Ref: No animal feces***	1.27	[0.90, 1.78]	0.17	1.84	[1.17, 2.89] *	0.01	1.26	[0.62, 2.56]	0.52	1.58	[0.68, 3.69]	0.29
**Animal Contact**												
Maternal-animal contact<br>***Ref: No contact***	1.00	[0.74, 1.37]	0.98	1.03	[0.65, 1.62]	0.11	1.08	[0.61, 1.90]	0.79	1.69	[0.89, 3.19]	0.90
Child-animal contact<br>***Ref: No contact***	1.29	[0.91, 1.82]	0.15	1.24	[0.72, 2.13]	0.45	0.65	[0.22, 1.87]	0.42	1.16	[0.42, 3.22]	0.77
**FECEZ-d scores**												
Medium scores<br>***Ref: Low score***	0.86	[0.61, 1.23]	0.42	0.97	[0.54, 1.73]	0.91	0.89	[0.49, 1.61]	0.69	1.91	[0.74, 4.91]	0.18
High score<br>***Ref: Low score***	1.05	[0.74, 1.49]	0.79	1.62	[0.92, 2.85]	0.09	0.83	[0.44, 1.59]	0.58	2.94	[1.17, 7.40] *	0.02

Table S5: Associations between animal-related variables and mean abundance of all animal MST markers. Mean differences (PRs), 95% confidence intervals (CIs), and p-values were calculated using a generalized linear (Gaussian) regression model. P-values are based on z-statistics computed as the coefficient divided by its robust standard error, assuming a normal distribution.

**Animal-related Variables**	**Floors**			**Child hands**			**Maternal hands**		
	**Mean diff.**	**[95% CIs]**	**p-value**	**Mean diff.**	**[95% CIs]**	**p-value**	**Mean diff.**	**[95% CIs]**	**p-value**
**Animal Ownership**									
Animals spend time outside HH<br>***Ref: Animals do not spend time outside***	-0.04	[-0.14, 0.06]	0.45	0.01	[-0.09, 0.10]	0.89	0.03	[-0.02, 0.08]	0.21
Animals spend time inside HH<br>***Ref: Animals do not spend time outside***	-0.06	[-0.17, 0.05]	0.30	0.01	[-0.09, 0.12]	0.79	0.03	[-0.02, 0.09]	0.27
Animals owned (>5)<br>***Ref: No animals owned***	-0.01	[-0.14, 0.13]	0.94	0.04	[-0.09, 0.17]	0.55	0.05	[-0.02, 0.12]	0.14
Animals owned (1-5)<br>***Ref: No animals owned***	-0.02	[-0.12, 0.09]	0.73	0.02	[-0.07, 0.12]	0.66	0.03	[-0.02, 0.08]	0.31
Animals owned<br>***Ref: No animals owned***	-0.01	[-0.11, 0.08]	0.76	0.03	[-0.06, 0.12]	0.54	0.03	[-0.01, 0.08]	0.15
**Animal Feces**									
Animal feces-Other management<br>***Ref: No animal feces***	0.20	[0.02, 0.37] *	0.03	0.02	[-0.14, 0.18]	0.81	-0.04	[-0.13, 0.05]	0.38
Animal feces left sometime/not removed<br>***Ref: No animal feces***	0.04	[-0.11, 0.19]	0.62	-0.04	[-0.19, 0.10]	0.55	0.02	[-0.06, 0.09]	0.67
Animal feces removed as soon as seen<br>***Ref: No animal feces***	0.00	[-0.12, 0.11]	0.93	-0.06	[-0.17, 0.04]	0.24	-0.01	[-0.06, 0.05]	0.80
Animal feces outside HH<br>***Ref: No animal feces***	0.04	[-0.05, 0.14]	0.37	-0.04	[-0.13, 0.04]	0.33	-0.01	[-0.05, 0.04]	0.76
Animal feces inside HH<br>***Ref: No animal feces***	-0.09	[-0.25, 0.07]	0.25	0.27	[0.12, 0.41] *	0.00	0.10	[0.02, 0.18] *	0.01
**Animal Contact**									
Maternal-animal contact<br>***Ref: No contact***	0.01	[-0.10, 0.11]	0.87	0.12	[0.02, 0.21] *	0.02	0.03	[-0.02, 0.08]	0.26
Child-animal contact<br>***Ref: No contact***	0.05	[-0.10, 0.21]	0.48	0.04	[-0.10, 0.19]	0.55	-0.02	[-0.09, 0.06]	0.69
**FECEZ-d scores**									
Medium scores<br>***Ref: Low score***	0.02	[-0.11, 0.14]	0.79	-0.01	[-0.12, 0.11]	0.91	0.02	[-0.04, 0.08]	0.44
High score<br>***Ref: Low score***	0.07	[-0.06, 0.20]	0.29	0.09	[-0.03, 0.20]	0.15	0.06	[0.00, 0.12]	0.07

Table S6: Associations between household and WASH-related variables and presence/absences of human HF183 MST marker. Prevalence ratios (PRs), 95% confidence intervals (CIs), and p-values were calculated using a modified Poisson regression model with a log link and robust standard errors. P-values are based on z-statistics computed as the coefficient divided by its robust standard error, assuming a normal distribution.

Household & WASH-related Variables	Any sample			Floors			Child hands			Maternal Hands		
	PR	[95% CIs]	p-value	PR	[95% CIs]	p-value	PR	[95% Cis]	p-value	PR	[95% Cis]	p-value
**Household factors**												
Unimproved floor<br>***Ref:*** *Improved floor*	0.94	[0.75, 1.16]	0.55	0.86	[0.60, 1.24]	0.42	0.94	[0.63, 1.40]	0.76	0.96	[0.66, 1.38]	0.81
Crowded<br>***Ref:*** *Uncrowded*	1.10	[0.85, 1.43]	0.45	1.45	[0.93, 2.27]	0.10	1.04	[0.57, 1.89]	0.91	1.39	[0.82, 2.37]	0.23
**Urbanicity**												
Rural<br>***Ref:*** *Urban*	0.92	[0.71, 1.19]	0.52	0.70	[0.44, 1.11]	0.13	1.18	[0.67, 2.08]	0.58	1.46	[0.74, 2.89]	0.27
Intermediate<br>***Ref:*** *Urban*	0.95	[0.73, 1.23]	0.68	0.90	[0.59, 1.37]	0.62	1.21	[0.69, 2.11]	0.51	1.23	[0.61, 2.46]	0.56
**Water**												
E. coli in drinking water<br>***Ref:*** *No E. coli in drinking water*	1.30	[1.00, 1.68] *	0.05	0.97	[0.66, 1.41]	0.86	1.67	[0.91, 3.04]	0.10	1.73	[0.92, 3.26]	0.09
Stored drinking water<br>***Ref:*** *Non stored water*	0.90	[0.74, 1.10]	0.30	0.73	[0.52, 1.04]	0.08	1.43	[0.88, 2.32]	0.15	0.78	[0.54, 1.12]	0.18
Unimproved drinking water source<br>***Ref:*** *Improved water source*	1.18	[0.88, 1.58]	0.26	1.04	[0.64, 1.69]	0.89	1.32	[0.83, 2.10]	0.24	0.97	[0.56, 1.69]	0.93
**Hygiene**												
Hygiene score (1-5 points)<br>***Ref:*** *Hygiene score (6-9 points)*	0.95	[0.78, 1.15]	0.59	0.86	[0.60, 1.22]	0.40	1.09	[0.74, 1.62]	0.65	0.74	[0.51, 1.07]	0.10
E. coli on maternal hands<br>***Ref:*** *Not E. coli in maternal hands*	1.00	[0.77, 1.28]	0.97	0.91	[0.57, 1.45]	0.69	0.88	[0.48, 1.63]	0.69	0.78	[0.44, 1.39]	0.40
E. coli on child hands<br>***Ref:*** *Nor E. coli in child hands*	1.21	[0.95, 1.53]	0.12	1.53	[1.04, 2.23] *	0.03	1.29	[0.80, 2.10]	0.30	1.32	[0.80, 2.19]	0.27
**Sanitation**												
Pit latrine<br>***Ref:*** *Flush to sewer*	1.27	[0.91, 1.78]	0.17	1.19	[0.65, 2.15]	0.57	1.78	[0.94, 3.38]	0.08	1.42	[0.72, 2.82]	0.31
Flush to septic tank<br>***Ref:*** *Flush to sewer*	1.19	[0.86, 1.64]	0.29	1.22	[0.74, 2.01]	0.44	1.27	[0.72, 2.27]	0.41	1.35	[0.73, 2.51]	0.34
Flush to another place/Open defecation<br>***Ref:*** *Flush to sewer*	1.03	[0.62, 1.69]	0.91	0.61	[0.26, 1.46]	0.27	1.02	[0.49, 2.14]	0.95	1.39	[0.62, 3.12]	0.42

Table S7: Associations between household and WASH-related variables and abundance of human HF183 MST marker. Mean differences (PRs), 95% confidence intervals (CIs), and p-values were calculated using a generalized linear (Gaussian) regression model. P-values are based on z-statistics computed as the coefficient divided by its robust standard error, assuming a normal distribution.

Household & WASH-related Variables	Floors			Child hands			Maternal hands		
	Mean diff.	[95% CIs]	p-value	Mean diff.	[95% CIs]	p-value	Mean diff.	[95% CIs]	p-value
**Household factors**									
Unimproved floor<br>***Ref:*** *Improved floor*	-0.21	[-0.47, 0.05]	0.11	-0.13	[-0.45, 0.19]	0.43	-0.07	[-0.41, 0.27]	0.68
Crowded<br>***Ref:*** *Uncrowded*	0.11	[-0.25, 0.47]	0.55	0.30	[-0.15, 0.75]	0.19	0.37	[-0.10, 0.84]	0.12
**Urbanicity**									
Rural<br>***Ref:*** *Urban*	-0.02	[-0.36, 0.32]	0.92	0.21	[-0.22, 0.63]	0.34	0.22	[-0.22, 0.66]	0.33
Intermediate<br>***Ref:*** *Urban*	0.14	[-0.20, 0.47]	0.43	0.24	[-0.18, 0.66]	0.26	0.17	[-0.27, 0.62]	0.44
**Water**									
E. coli in drinking water<br>***Ref:*** *No E. coli in drinking water*	0.03	[-0.26, 0.32]	0.84	0.31	[-0.05, 0.67]	0.09	0.02	[-0.36, 0.41]	0.91
Stored drinking water<br>***Ref:*** *Non stored water*	-0.17	[-0.42, 0.07]	0.17	-0.10	[-0.41, 0.21]	0.52	-0.06	[-0.39, 0.26]	0.70
Unimproved drinking water source<br>***Ref:*** *Improved water source*	-0.09	[-0.44, 0.26]	0.62	-0.03	[-0.47, 0.40]	0.88	0.24	[-0.21, 0.70]	0.29
**Hygiene**									
Hygiene score (1-5 points)<br>***Ref:*** *Hygiene score (6-9 points)*	-0.02	[-0.26, 0.22]	0.87	-0.11	[-0.41, 0.19]	0.47	-0.16	[-0.47, 0.15]	0.32
E. coli on maternal hands<br>***Ref:*** *Not E. coli in maternal hands*	0.10	[-0.24, 0.45]	0.56	-0.05	[-0.49, 0.38]	0.81	-0.30	[-0.75, 0.16]	0.20
E. coli on child hands<br>***Ref:*** *Nor E. coli in child hands*	0.30	[0.02, 0.57]*	0.03	0.22	[-0.12, 0.57]	0.21	0.03	[-0.33, 0.40]	0.87
**Sanitation**									
Pit latrine<br>***Ref:*** *Flush to sewer*	0.05	[-0.36, 0.47]	0.81	0.23	[-0.29, 0.75]	0.39	0.37	[-0.18, 0.92]	0.18
Flush to septic tank<br>***Ref:*** *Flush to sewer*	-0.04	[-0.38, 0.31]	0.84	-0.03	[-0.47, 0.40]	0.88	0.17	[-0.29, 0.62]	0.47
Flush to another place/Open defecation<br>***Ref:*** *Flush to sewer*	-0.23	[-0.71, 0.26]	0.36	-0.04	[-0.64, 0.57]	0.91	0.18	[-0.46, 0.82]	0.58

Figure S1: Known groups construct validity of the FECEZ-derived scores (FECEZ-d scores). Boxplots show total score (top row), environment sub-domain (middle row), and behavior sub-domain (bottom row) across community type, child age, and household animal ownership. Pairwise comparisons for community type and child age are Tukey-adjusted; animal ownership was compared using the Wilcoxon rank-sum test. ****p<0.0001; *p<0.05.

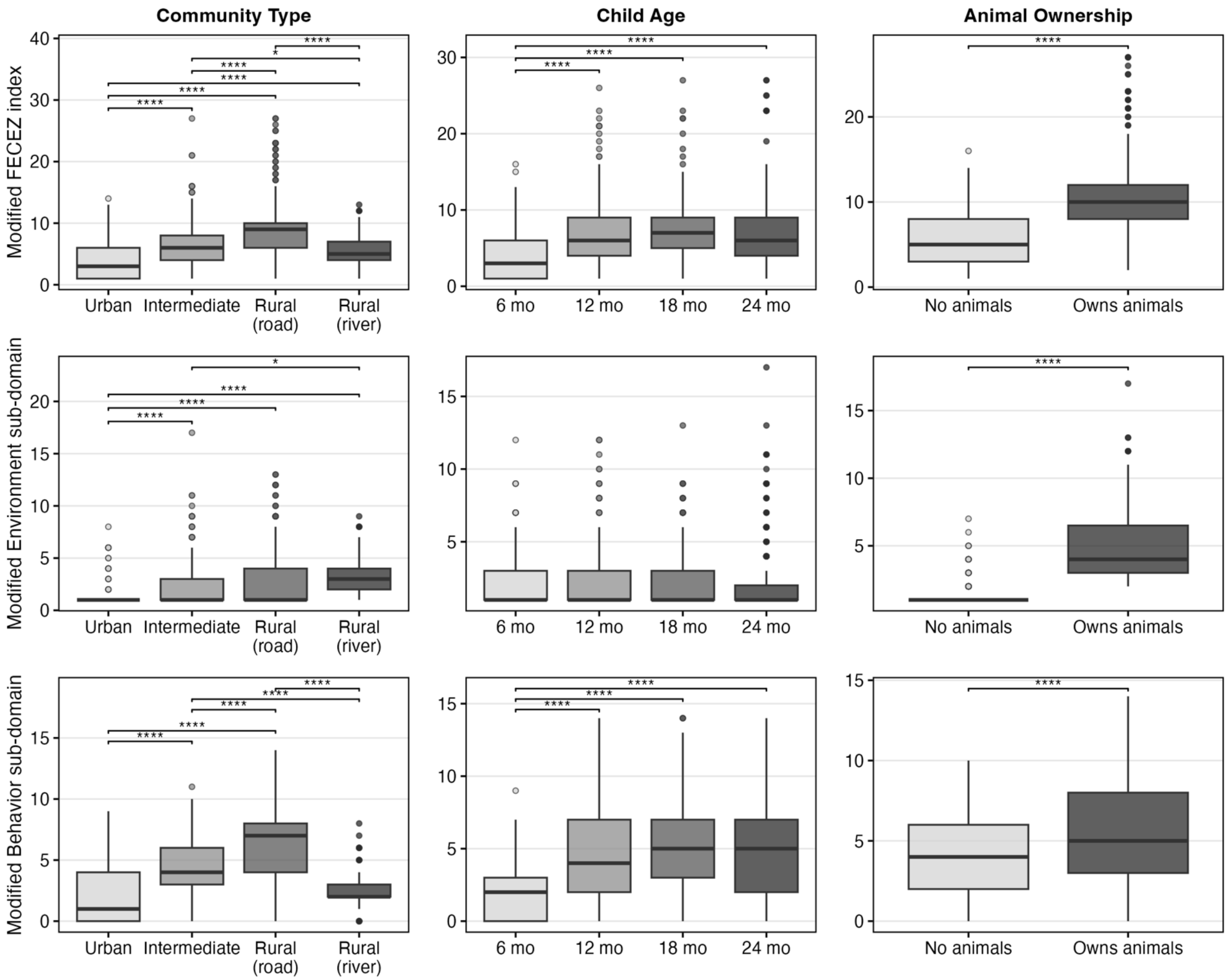

Figure S2. Directed acyclic graph (DAG). Illustrating the hypothesized relationships between environmental risk factors, covariates, and MST marker presence on floors, child hands, and maternal hands. The red-highlighted variables represent the minimal sufficient adjustment set required to block all backdoor pathways between exposure and outcome. SES: Socio-economic status.

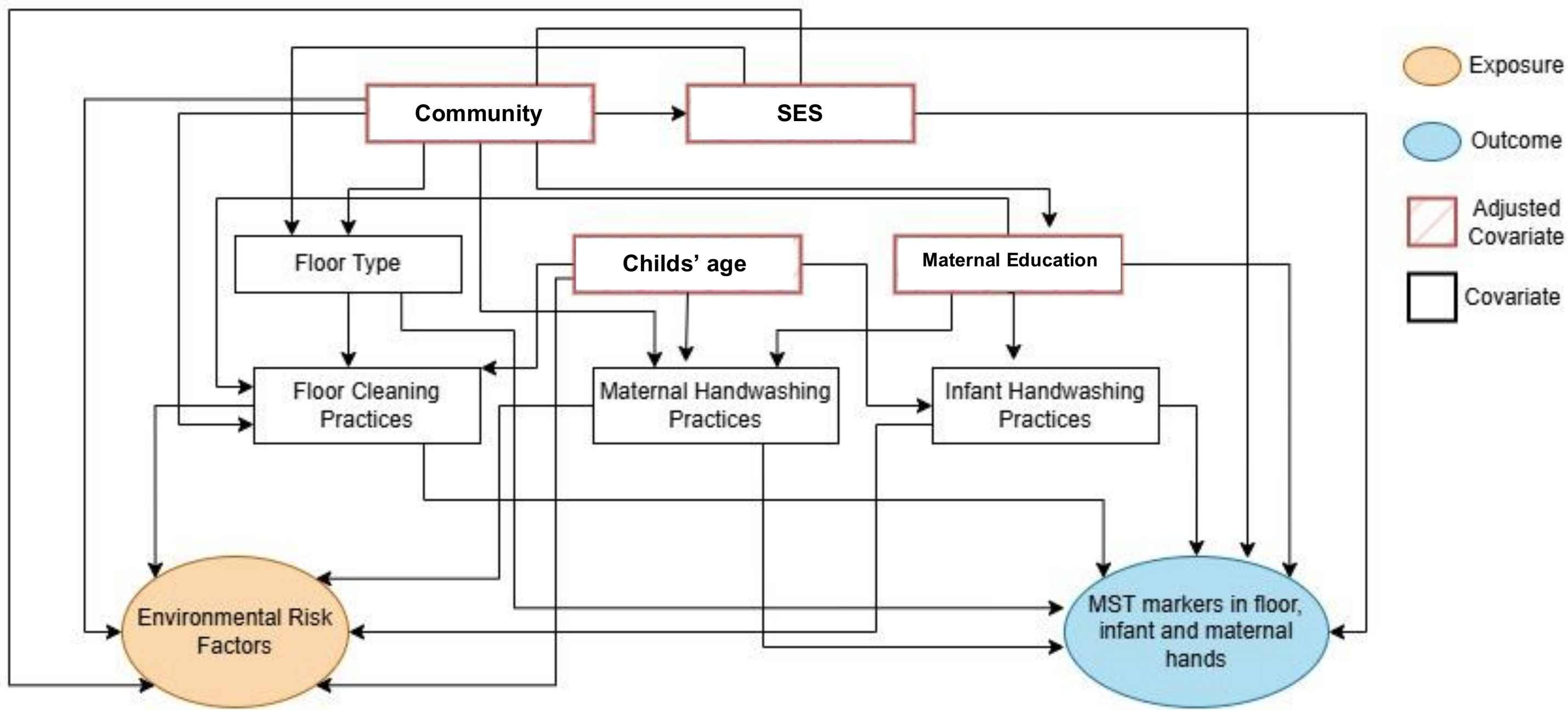

Figure S3: Canine and Avian MST markers (presence/absence) associations

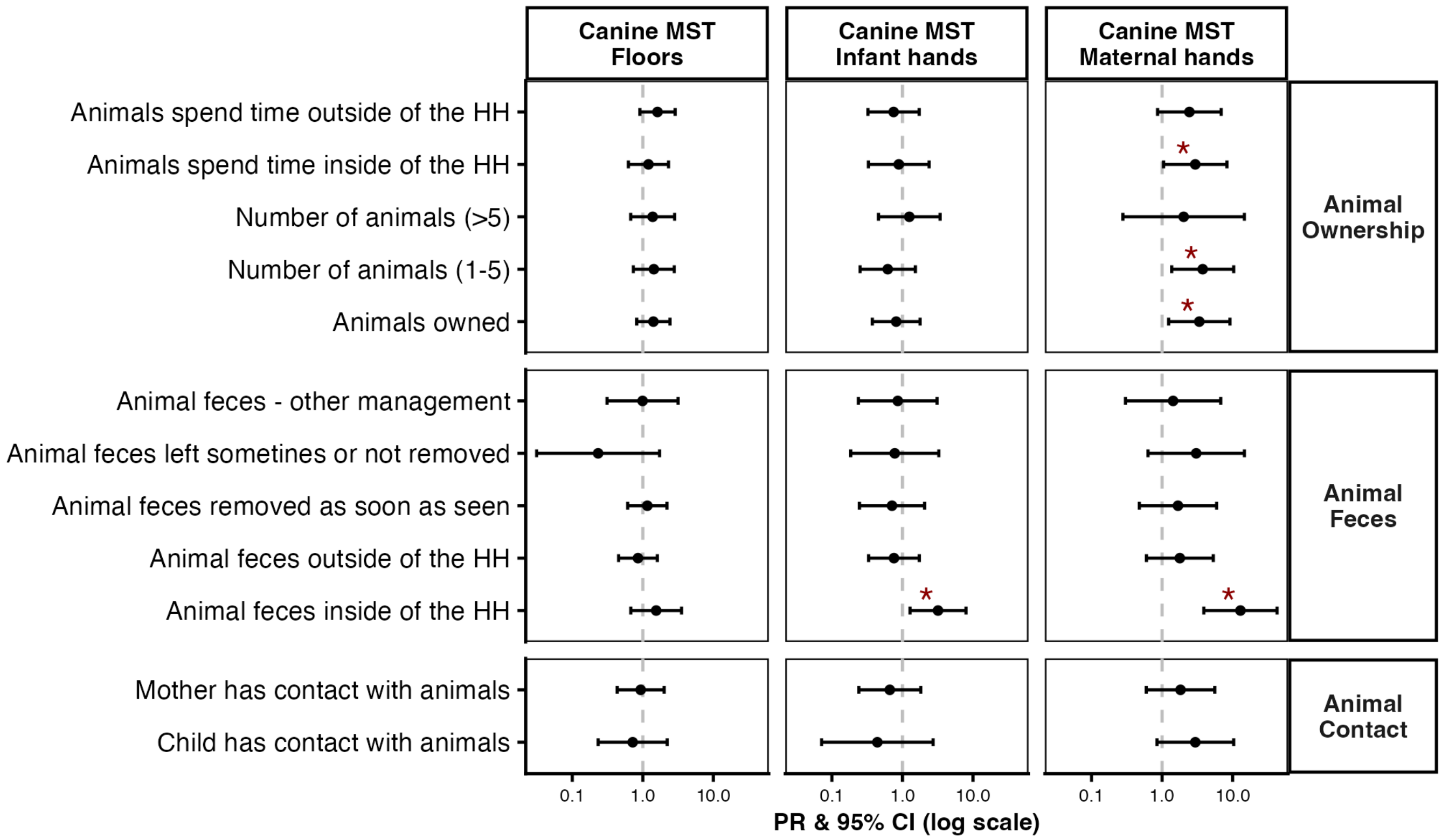

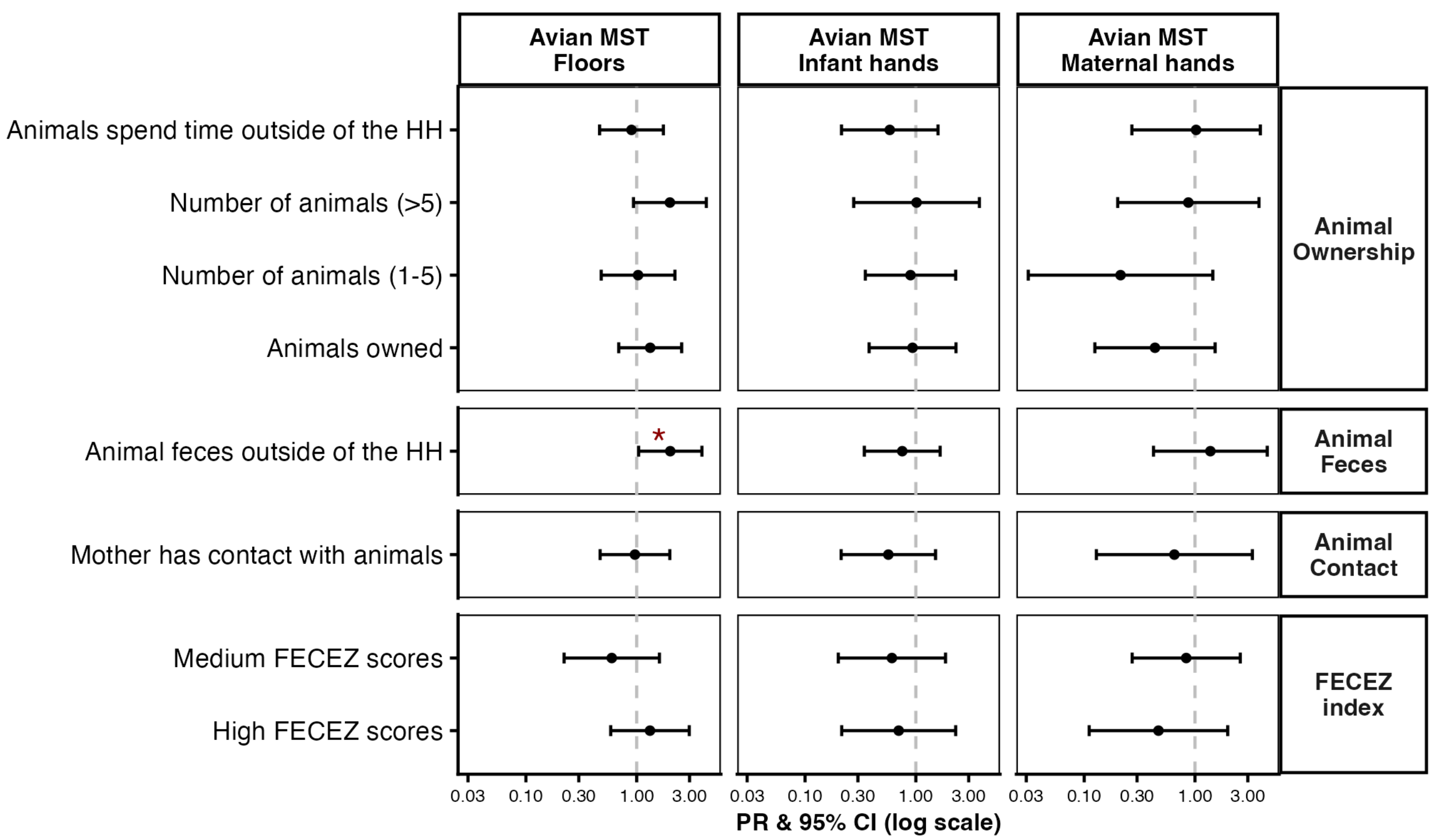

Figure S4: Canine and Avian MST markers (abundance) associations

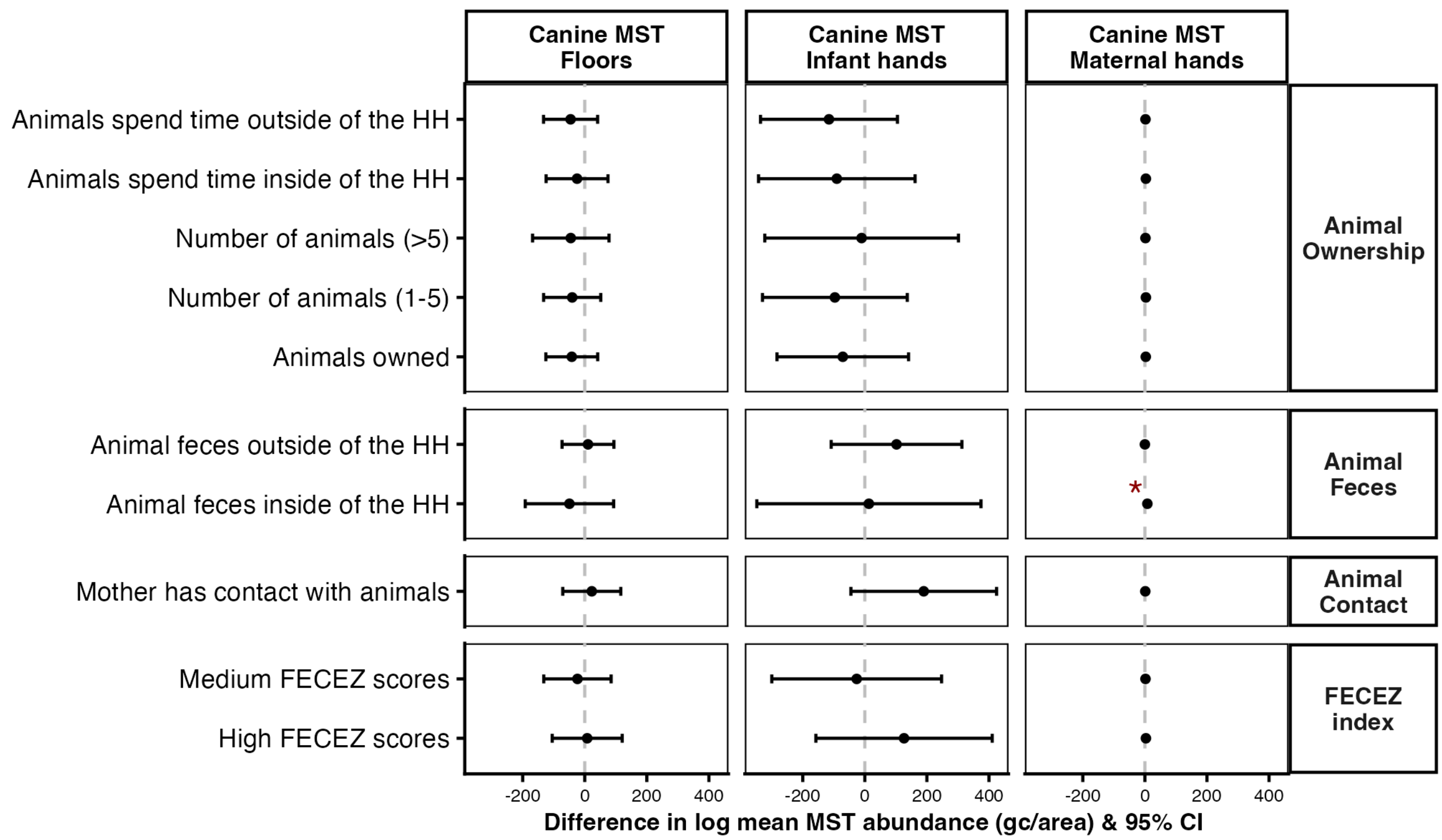

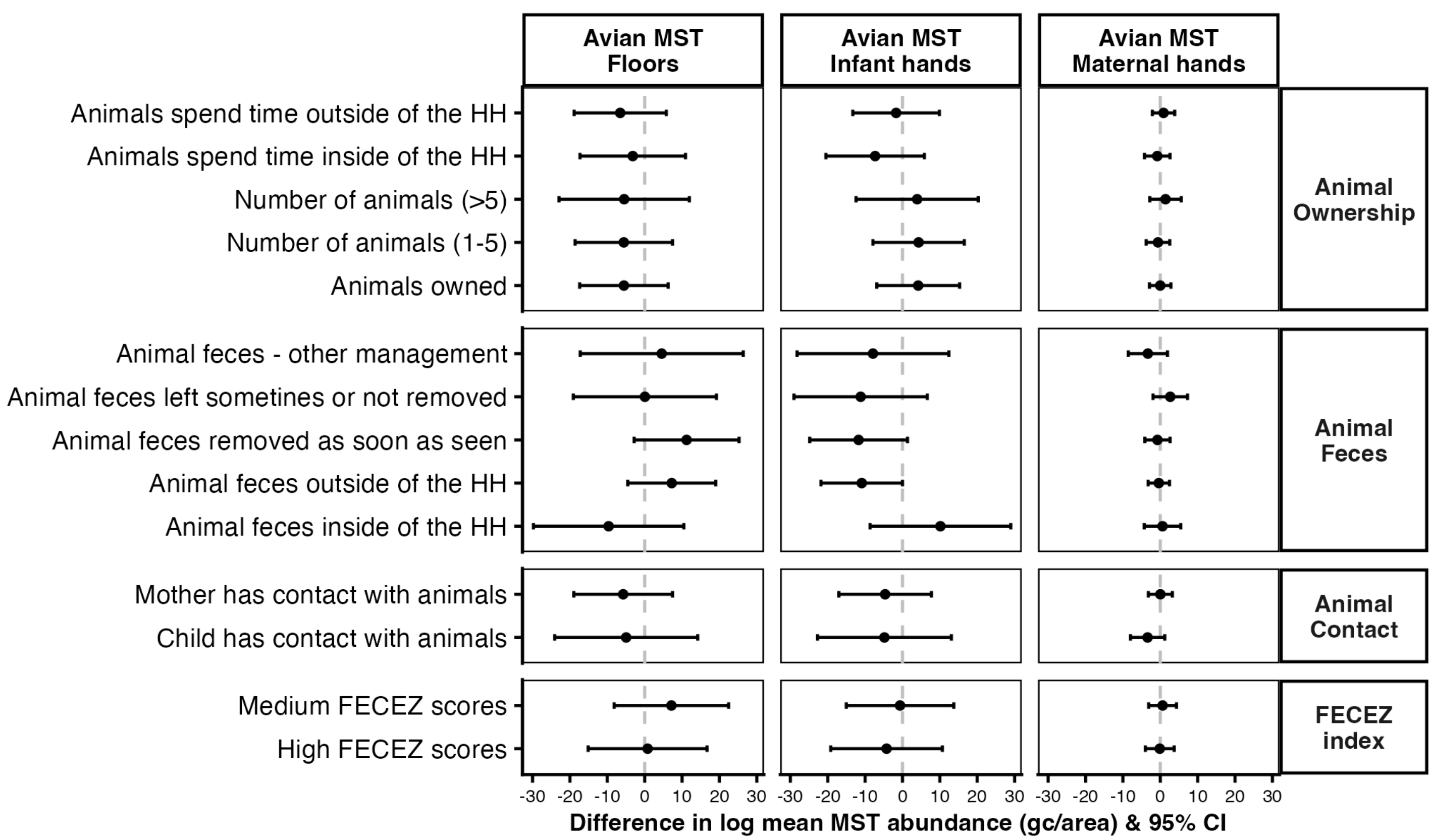

Supplement: Supplementary file 1 [file media-1.pdf]
